# Comparative dosimetric findings using accelerated partial breast irradiation across five catheter subtypes

**DOI:** 10.1186/s13014-015-0468-7

**Published:** 2015-07-31

**Authors:** Zaker Rana, Nadim M. Nasr, Huaying Ji, Virginia Lorio, Stephanie Akbari, Molly Sebastian, Mami Martin, Robert L. Hong

**Affiliations:** Department of Radiation Oncology, Virginia Hospital Center, 1701 N George Mason Dr, Arlington, VA 22205 USA

**Keywords:** APBI, brachytherapy, Mammosite, Contura, Savi

## Abstract

**Purpose:**

Accelerated partial breast irradiation (APBI) with balloon and strut adjusted volume implants (SAVI) show promising results with excellent tumor control and minimal toxicity. Knowing the factors that contribute to a high skin dose, rib dose, and D_95_ coverage may reduce toxicity, improve tumor control, and help properly predict patient outcomes following APBI.

**Methods and materials:**

A retrospective analysis of 594 patients treated with brachytherapy based APBI at a single institution from May 2008 to September 2014 was grouped by applicator subtype. Patients were treated to a total of 34 Gy (3.4 Gy x 10 fractions over 5 days delivered BID) targeting a planning target volume (PTV) 1.0 cm beyond the lumpectomy cavity using a high dose rate source.

**Results:**

SAVI devices had the lowest statistically significant values of D_max_Skin (81.00 ± 29.83), highest values of D_90_ (101.50 ± 3.66), and D_95_ (96.09 ± 4.55). SAVI-mini devices had the lowest statistically significant values of D_max_Rib (77.66 ± 32.92) and smallest V_150_ (18.01 ± 3.39). Multi-lumen balloons were able to obtain the smallest V_200_ (5.89 ± 2.21). Strut-based applicators were more likely to achieve a D_max_Skin and a D_max_Rib less than or equal to 100 %. The effect of PTV on V_150_ showed a strong positive relationship (*p* < .001). PTV and D_max_Skin showed a weak negative relationship in multi-lumen applicators (*p* = .016) and SAVI-mini devices (*p* < .001). PTV and D_max_Rib showed a weak negative relationship in multi-lumen applicators (*p* = .009), SAVI devices (*p* < .001), and SAVI-mini devices (*p* < .001).

**Conclusion:**

PTV volume is strongly correlated with V_150_ in all devices and V_200_ in strut based devices. Larger PTV volumes result in greater V_150_ and V_200_, which could help predict potential risks for hotspots and resulting toxicities in these devices. PTV volume is also weakly negatively correlated with max skin dose and max rib dose, meaning that as the PTV volumes increase one can expect slightly smaller max skin and rib doses. Strut based applicators are significantly more effective in keeping skin and rib dose constraints under 125 and 100 % when compared to any balloon based applicator.

## Background

Accelerated partial breast irradiation (APBI) has gained popularity as an alternative option to deliver adjuvant radiation therapy (RT) after lumpectomy in select patient populations with early stage breast cancer [1]. Several different forms of RT can be used to deliver APBI, including interstitial multicatheter brachytherapy, balloon catheter brachytherapy, intraoperative radiation therapy, and conformal external beam radiation therapy [2]. Initial data has been reported using interstitial multicatheter brachytherapy, a technique that uses image guidance for the insertion of multiple afterloading catheters around the lumpectomy cavity, resulting in excellent target coverage and conformality [3–6]. Despite 10-year interstitial multicatheter brachytherapy results showing a local control rate ≥ 95 %, and excellent cosmetic outcomes in 90 % of patients, physician acceptance has been slow [7–10, 6]. The slow acceptance is partly due to the high degree of technical skill required for a successful interstitial implant [3, 11].

The MammoSite Single-Lumen (MS-SL) applicator (Hologic Inc, Bedford, MA) was introduced to simplify application and make results reproducible. The MammoSite catheter is composed of a 15 cm double-lumen catheter, that is 6 mm in diameter, and connected to a silicone balloon. The balloon is inflated to a size that completely fills the lumpectomy cavity and the prescription radiation dose is inserted through the catheter into the balloon [2]. A minimum balloon-to-skin distance of 5 mm is required with a threshold of at least 7 mm strongly recommended, as longer skin distance is associated with improved cosmesis [12–14]. MammoSite has been shown to be effective with low local recurrence rates and toxicity rates in both single institutional experiences and large multi-institutional experiences, like the American Society of Breast Surgeons MammoSite registry trial [15, 16]. Patients with small breasts or upper-inner quadrant tumors are not eligible for MS-SL due to the balloon surface being too close to the skin. Furthermore, since the device contains a single central source channel, geometry is fixed and dose optimization is limited [17, 18].

In order to eliminate dosimetric limitations seen in single lumen devices, Hologic introduced a MammoSite Multi-lumen (MS-ML) device and the Contura Multi-Lumen Balloon (Bard Biopsy Systems, Tempe, AZ) was developed. The MS-ML device has fewer outer lumens (3 vs. 4) with a shorter offset (3 mm vs. 5 mm) from the central lumen when compared with the Contura device, but the two devices have been shown to produce a clinically comparable plan [19]. The outer lumens provide additional source positions and better dose flexibility when compared with a single-catheter approach. Multi-lumen catheters have also shown improvements in rib doses, skin doses, and PTV-EVAL coverage, when compared to single-lumen devices [20].

While multicatheter brachytherapy provides superior versatility and dosimetric conformity, they also require multiple skin incisions for placement. Hybrid brachytherapy devices were developed to provide dosimetric advantages while maintaining the simplicity and aesthetics of single-catheter breast brachytherapy devices [2]. The Strut Adjusted Volume Implant (SAVI) device (Cianna Medical, AlisoViejo, CA) is placed into a lumpectomy cavity through a single incision. The SAVI applicator itself has a central catheter as well as 6, 8, or 10 peripheral catheters that are expanded outwards to the periphery of the lumpectomy cavity after insertion [21]. The central and peripheral catheters contain a large number of potential dwell positions for the radiation source and are in direct contact with the lumpectomy cavity edge, providing flexibility in dose distribution [22].

APBI using balloon and strut-based applicators show promising results with excellent tumor control and minimal toxicity [23]. A higher dose to 95 % of the planning target volume (D95) is important in achieving tumor control [24]. Achieving better cosmetic outcomes and reducing toxicity requires reduction in normal tissue exposure. Telangiectasia development has been shown to be a function of skin dose. The Virginia Commonwealth University experience, Contura phase IV registry trial, and recommendations in the ongoing NSABP-B39 protocol proposed skin consraints of ≤120, ≤125, and ≤145 % [25, 3]. However, recently published data suggest that skin doses ≥ 100 % may represent a stronger predictor of late telangiectasia [23].

Knowing the factors that contribute to a high skin dose, rib dose, V150, V200, and D95 coverage may reduce toxicity, improve tumor control, and help properly predict patient outcomes following APBI. We present here our single-institution dosimetric performance with Mammosite, Contura, and SAVI APBI. We further characterize dosimetric correlates to reduce toxicity and maximize cosmetic preservation.

## Methods and materials

A total of 594 patients, with localized breast cancer treated with brachytherapy based APBI at a single institution from May 2008 to September 2014, were retrospectively reviewed as part of a prospectively maintained institutional database. Due to the retrospective nature of this study, it was granted an exemption in writing by the Virginia Hospital Center IRB. Of the 594 patients, there were 496 strut based implants, including SAVI 6-1 mini, 6-1, 8-1, and 10-1 devices, but because of SAVI 6-1 mini’s unique size it was separated into its own group. Multi-catheter balloon implants consisted of 54 Contura devices and 10 MammoSite mulit-lumen devices. Because the two devices have been shown to produce similar plans, they were combined into one multi-lumen balloon subgroup. Categories used for statistical analysis included 243 SAVI devices, 253 SAVI 6-1 mini devices, 64 multi-lumen balloons, and 34 Mammosite single-lumen balloons.

Generally inclusion criteria for APBI were in accordance with the American Society of Breast Surgeons (ASBS) and the American Society for Radiation Oncology (ASTRO): invasive carcinoma or ductal carcinoma in situ, tumor <3 cm, negative microscopic surgical margins, negative lymph nodes, and age 45 or older.

Patients were treated to a total of 34 Gy (3.4 Gy x 10 fractions over 5 days delivered BID) targeting a PTV 1 cm beyond the lumpectomy cavity using a high dose rate source. Minimum treatment planning goals for the planning target volume were initially D90 > 90 %; in October 2011, PTV coverage goals were adjusted to D_95_ > 95 %. Size of the planning target volume, absolute volume of the tissue receiving 150 % of the prescription dose (V_150_), and volume of tissue receiving 200 % (V200), were evaluated. 3D treatment planning system was used to obtain the maximal point doses delivered to the skin and chest wall.

Statistical analysis was performed with SPSS v22.0 (SPSS Inc, Chicago, IL). Statistical significance was defined as *p* < .05. Multiple analysis-of-variance (MANOVA) was performed to search for possible differences between catheter subtypes. Dosimetric parameters were then compared across catheter subtype using Student’s *t*-test (*α* < 0.05). Multiple logistic regression with backward elimination was used in the multivariate analysis to search for possible predicting factors for maximum skin dose, maximum rib dose, and D95. Pearson’s correlation coefficient was used to see PTV’s effect on D_90_, D_95_, V_150_, V_200_, D_max_Skin, and D_max_Rib. Specifically, the basis of this analysis was to determine if larger PTV volumes would cause an increase in maximum doses and potential toxicities. A strong relationship was defined as an r-value greater than 0.5 or less than−0.5 with *p* < .05. A weak relationship was defined as an r-value between 0.25 and 0.5 or−0.25 and−0.5 with *p* < .05.

## Results

**Fig. 1:**
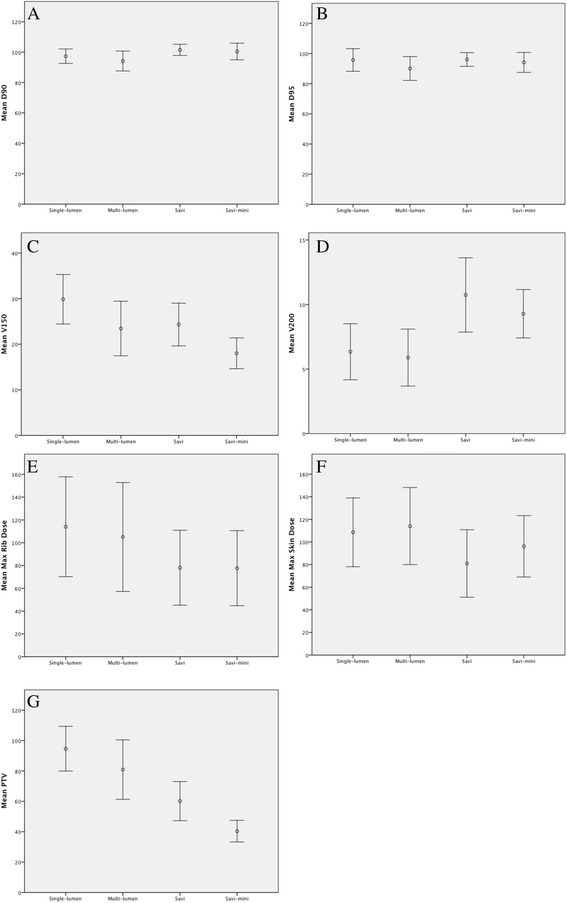
Mean dosimetric values +/−one standard deviation across APBI device (**a**) D_90_ (**b**) D_95_ (**c**) V_150_ (**d**) V_200_ (**e**) D_max_Rib (**f**) D_max_Skin (**g**) PTV

The baseline characteristics for included patients (*n* = 594) are shown in Table 1. The mean values with the standard deviations of achieved dosimetric characteristics are shown in Table 2 and Fig. 1. Student’s *t*-test was used to compare the dosimetric characteristics across each type of APBI device (Tables 3, 4 and 5).

**Table 1:** Baseline patient treatment characteristics

		Patients *(N = 594)*
Age (y/o)	Median 63 (37–92)	
	>60, n (%)	342 (57.6 %)
	50–60, n (%)	173 (29.1 %)
	>40–50, n (%)	78 (13.1 %)
	<40, n (%)	1 (0.2 %)
Tumor location, n (%)	Left breast	297 (50.0 %)
	Right breast	297 (50.0 %)
Tumor Size (mm)	Median	13
	Range	2–25
Grade	Median	2
	Range	1–3

**Table 2:** Dose distribution parameters (mean values and standard deviation) across catheter subtype

	Single-lumen (*n* = 34)	Multi-lumen (*n* = 64)	SAVI (*n* = 243)	SAVI-mini (*n* = 253)	All Devices (*n* = 594)
D_90_ (%)	97.33 ± 4.75	94.16 ± 6.54	101.50 ± 3.66	100.45 ± 5.48	100.02 ± 5.40
D_95_ (%)	95.74 ± 7.47	90.06 ± 7.90	96.09 ± 4.55	94.12 ± 6.61	94.58 ± 6.33
D_max_Skin	108.56 ± 30.44	114.09 ± 34.10	81.00 ± 29.83	96.20 ± 27.17	92.62 ± 31.26
D_max_Rib	114 ± 43.82	105.03 ± 47.80	78.06 ± 32.87	77.66 ± 32.92	82.85 ± 37.16
V_150_ (cm^3^)	29.86 ± 5.44	23.44 ± 5.98	24.32 ± 4.69	18.01 ± 3.39	21.85 ± 5.66
V_200_ (cm^3^)	6.34 ± 2.17	5.89 ± 2.21	10.74 ± 2.88	9.28 ± 1.87	9.34 ± 2.87
PTV (cm^3^)	94.62 ± 14.72	80.88 ± 19.55	60.15 ± 12.89	40.36 ± 7.13	55.93 ± 20.14

**Table 3:** Comparison of skin and rib doses between APBI devices

Dependent Variable	(I) Applicator	(J) Applicator	Mean Difference (I–J)	Std. Error	*p*
D_max_Skin	Single-lumen	Multi-lumen	−5.5242	6.20960	.374
		Savi	27.5618	5.35769	< .001
		Savi-mini	12.3611	5.34468	.021
	Multi-lumen	Single-lumen	5.5242	6.20960	.374
		Savi	33.0860	4.11108	< .001
		Savi-mini	17.8853	4.09411	< .001
	Savi	Single-lumen	−27.5618	5.35769	< .001
		Multi-lumen	−33.0860	4.11108	< .001
		Savi-mini	−15.2007	2.62820	< .001
	Savi-mini	Single-lumen	−12.3611	5.34468	.021
		Multi-lumen	−17.8853	4.09411	< .001
		Savi	15.2007	2.62820	< .001
D_max_Rib	Single-lumen	Multi-lumen	8.9750	7.52486	.233
		Savi	35.9376	6.49250	< .001
		Savi-mini	36.3416	6.47673	< .001
	Multi-lumen	Single-lumen	−8.9750	7.52486	.233
		Savi	26.9626	4.98185	< .001
		Savi-mini	27.3666	4.96129	< .001
	Savi	Single-lumen	−35.9376	6.49250	< .001
		Multi-lumen	−26.9626	4.98185	< .001
		Savi-mini	.4040	3.18487	.899
	Savi-mini	Single-lumen	−36.3416	6.47673	< .001
		Multi-lumen	−27.3666	4.96129	< .001
		Savi	−.4040	3.18487	.899

**Table 4:** Comparison of D_90_ and D_95_ between APBI devices

	(I) Applicator	(J) Applicator	Mean Difference (I–J)	Std. Error	*p*
D_90_	Single-lumen	Multi-lumen	3.1699	1.04264	.002
		Savi	−4.1677	.89960	< .001
		Savi-mini	−3.1167	.89741	.001
	Multi-lumen	Single-lumen	−3.1699	1.04264	.002
		Savi	−7.3376	.69028	< .001
		Savi-mini	−6.2866	.68743	< .001
	Savi	Single-lumen	4.1677	.89960	< .001
		Multi-lumen	7.3376	.69028	< .001
		Savi-mini	1.0510	.44129	.018
	Savi-mini	Single-lumen	3.1167	.89741	.001
		Multi-lumen	6.2866	.68743	< .001
		Savi	−1.0510	.44129	.018
D_95_	Single-lumen	Multi-lumen	5.6730	1.29051	< .001
		Savi	−.3506	1.11346	.753
		Savi-mini	1.6171	1.11076	.146
	Multi-lumen	Single-lumen	−5.6730	1.29051	< .001
		Savi	−6.0236	.85439	< .001
		Savi-mini	−4.0559	.85086	< .001
	Savi	Single-lumen	.3506	1.11346	.753
		Multi-lumen	6.0236	.85439	< .001
		Savi-mini	1.9677	.54620	< .001
	Savi-mini	Single-lumen	−1.6171	1.11076	.146
		Multi-lumen	4.0559	.85086	< .001
		Savi	−1.9677	.54620	< .001

**Table 5:** Comparison of PTV, V150, and V200 between APBI devices

	(I) Applicator	(J) Applicator	Mean Difference (I–J)	Std. Error	*p*
PTV	Single-lumen	Multi-lumen	13.7353	2.53559	< .001
		Savi	34.4664	2.18772	< .001
		Savi-mini	54.2576	2.18241	< .001
		Multi-lumen	13.7353	2.53559	< .001
	Multi-lumen	Single-lumen	−13.7353	2.53559	< .001
		Savi	20.7310	1.67869	< .001
		Savi-mini	40.5222	1.67176	< .001
	Savi	Single-lumen	−34.4664	2.18772	< .001
		Multi-lumen	−20.7310	1.67869	< .001
		Savi-mini	19.7912	1.07318	< .001
	Savi-mini	Single-lumen	−54.2576	2.18241	< .001
		Multi-lumen	−40.5222	1.67176	< .001
		Savi	−19.7912	1.07318	< .001
V_150_	Single-lumen	Multi-lumen	6.4121	.93452	< .001
		Savi	5.5340	.80631	< .001
		Savi-mini	11.8479	.80435	< .001
	Multi-lumen	Single-lumen	−6.4121	.93452	< .001
		Savi	−.8782	.61870	.156
		Savi-mini	5.4357	.61615	< .001
	Savi	Single-lumen	−5.5340	.80631	< .001
		Multi-lumen	.8782	.61870	.156
		Savi-mini	6.3139	.39553	< .001
	Savi-mini	Single-lumen	−11.8479	.80435	< .001
		Multi-lumen	−5.4357	.61615	< .001
		Savi	−6.3139	.39553	< .001
V_200_	Single-lumen	Multi-lumen	.4523	.50586	.372
		Savi	−4.4003	.43646	< .001
		Savi-mini	−2.9443	.43540	< .001
	Multi-lumen	Single-lumen	−.4523	.50586	.372
		Savi	−4.8526	.33490	< .001
		Savi-mini	−3.3966	.33352	< .001
	Savi	Single-lumen	4.4003	.43646	< .001
		Multi-lumen	4.8526	.33490	< .001
		Savi-mini	1.4560	.21410	< .001
	Savi-mini	Single-lumen	2.9443	.43540	< .001
		Multi-lumen	3.3966	.33352	< .001
		Savi	−1.4560	.21410	< .001

### D_max_Skin

**Fig. 2:**
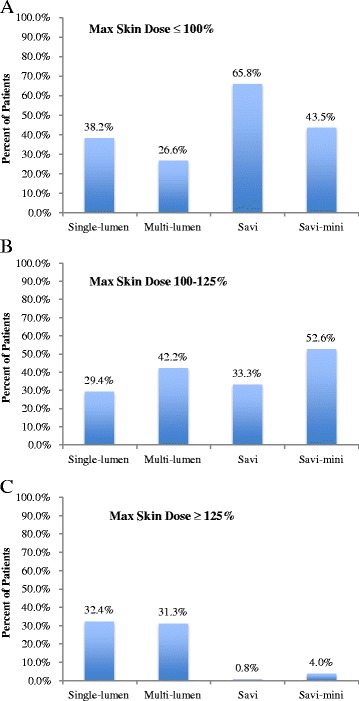
Percent of patients within a D_max_Skin range across applicator subtype (**a**) D_max_Skin ≤ 100 % (**b**) D_max_Skin 100–125 % (**c**) D_max_Skin ≥ 125 %

The lowest values of D_max_Skin were obtained in the SAVI devices (81.00 ± 29.83). This mean difference was significantly less than single-lumen balloon applicators (−27.56, *p* < .001), multi-lumen balloon applicators (−33.09, *p* < .001), and SAVI-mini devices (−15.20, *p* < .001) (Table 3). Strut-based applicators (SAVI: 0.8 %, SAVI-mini: 4.0 %) were much less likely to receive a D_max_Skin greater than or equal to 125 %, when compared to single-lumen (32.4 %) and multi-lumen balloon applicators (31.3 %) (Fig. 2). Strut-based applicators were also more likely to achieve a D_max_Skin less than or equal to 100 % with SAVI devices achieving this 65.8 % of the time.

### D_max_ Rib

**Fig. 3:**
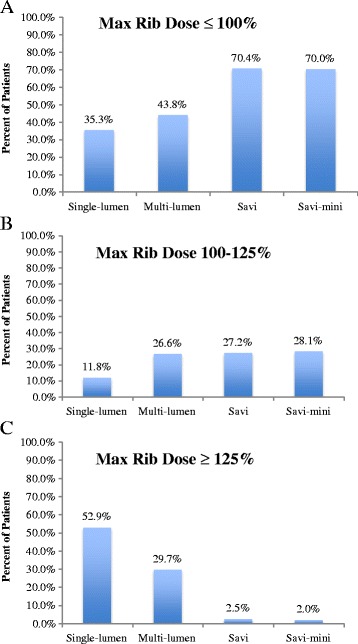
Percent of patients within a D_max_Rib range across applicator subtype (**a**) D_max_Rib ≤ 100 % (**b**) D_max_Rib 100–125 % (**c**) D_max_Rib ≥ 125 %

The lowest values of D_max_ Rib were seen in the SAVI-mini devices (77.66 ± 32.92). The mean difference in SAVI-mini devices was statistically significant when compared to single-lumen balloon applicators (−36.34, *p* < .001) and multi-lumen balloon applicators (−27.37, *p* < .001). There was no statistically significant difference in D_max_Rib when comparing SAVI-mini devices with SAVI devices. Strut-based devices were able to achieve a D_max_Rib less than or equal to 100 % in 70 % of patients treated, which was more frequent than the single-lumen balloon applicators (35.3 %) and multi-lumen balloon applicators (43.8 %) (Fig. 3).

### D_90_ and D_95_

SAVI devices had the highest values of D_90_ (101.50 ± 3.66) and D_95_ (96.09 ± 4.55). When compared to multi-lumen balloon applicators, strut-based devices had a statistically significant greater D_90_ mean difference (7.34, *p* < .001) and D_95_ mean difference (6.02, *p* < .001) (Table 4). However, the difference was not statistically significant different when compared to single-lumen devices.

### V_150_ and V_200_

SAVI-mini devices obtained the smallest V_150_ (18.01 ± 3.39) and this mean difference was significantly smaller than single-lumen (−11.85, *p* < .001), multi-lumen (−5.44, *p* < .001), and SAVI devices (−6.31, *p* < .001) (Table 5). When looking at V_200_, multi-lumen balloons were able to obtain the smallest volume (5.89 ± 2.21). This volume was significantly smaller than SAVI (−4.85, *p* < .001) and SAVI-mini devices (−3.40, *p* < .001), but when comparing V_200_ obtained in multi-lumen and single-lumen applicators there was no significant difference.

### PTV

PTV was statistically significantly smaller when SAVI and SAVI-mini devices were used as opposed to single-lumen and multi-lumen balloon applicators (Tables 2 and 5). The effect of PTV on dosimetric characteristics was observed using Pearson’s correlation coefficient in order to determine if larger PTV volumes resulted in greater maximum doses. When looking at all the devices together (*n* = 594), V_150_ was the only dosimetric constraint to show a strong positive relationship with PTV (*r* = .783, *p* < .001) (Table 6). This strong positive relationship between PTV and V_150_ was also observed when devices were stratified between SAVI (*r* = .808, *p* < .001), SAVI-mini devices (*r* = .826, *p* < .001), multi-lumen applicators (*r* = .547, *p* < .001), and single-lumen applicators (*r* = .513, *p* < .001). PTV and V_200_ showed a strong positive relationship in SAVI-mini devices (*r* = .586, *p* < .001) and a weak positive relationship in SAVI devices (*r* = .266, *p* < .001). PTV and D_max_Skin showed a weak negative relationship in multi-lumen applicators (*r* = -.301, *p* = .016) and SAVI-mini devices (*r* =−.350, *p* < .001). PTV and D_max_Rib showed a weak negative relationship in multi-lumen applicators (*r* =−.325, *p* = .009), SAVI devices (*r* = .407, *p* < .001), and SAVI-mini devices (*r* =−.297, *p* < .001). PTV had a weak positive relationship with D_90_ in multi-lumen balloon applicators (*r* = .388, *p* = .002) and SAVI-mini devices (*r* = .335, *p* < .001). PTV also had a weak positive relationship with D_95_ in multi-lumen balloon applicators (*r* = .382, *p* = .002) and SAVI-mini devices (*r* = .405, *p* < .001).

**Table 6:** The Effect of PTV on D_90_, D_95_, D_max_Skin, D_max_Rib, V_150_, and V_200_

	Single-lumen		Multi-lumen		SAVI		SAVI 6-1 mini		Total	
	(*n* = 34)		(*n* = 64)		(*n* = 243)		(*n* = 253)		(*n* = 594)	
	*r*	*p*	*r*	*p*	*r*	*p*	*r*	*p*	*r*	*p*
PTV (cc) vs. D_90_	.236	.180	.388*	.002	.072	.263	.335*	< .001	−.082*	.046
PTV (cc) vs D_95_	.102	.565	.382*	.002	.129*	.044	.405*	< .001	.111*	.007
PTV (cc) vs D_max_Skin	−.153	.388	−.301*	.016	−.056	.384	−.350*	< .001	−.021	.603
PTV (cc) vs D_max_Rib	−.217	.217	−.325*	.009	−.407*	< .001	−.297*	< .001	.020	.626
PTV (cc) vs V_150_	.513*	.002	.547*	< .001	.808*	< .001	.826*	< .001	.783*	< .001
PTV (cc) vs V_200_	−.332	.055	−.176	.163	.266*	< .001	.586*	< .001	−.125*	.002

Any relationship is demarcated with *. A strong relationship was defined as an r-value greater than 0.5 or less than −0.5 with p < .05. A weak relationship was defined as an r-value between 0.25 and 0.5 or −0.25 and −0.5 with p < .05

Of the 594 patients treated, 139 of the patients were followed for three years after treatment. There were 3 local failures (2.2 %) after three years resulting in a local control rate of 97.8 %. There were a total of 2 contralateral failures (1.4 %). Two and three year disease free survival rate were 98.8 and 96.3 % respectively.

## Discussion

Our current report documents the ability to achieve dosimetric prescription goals across various applicators in patients treated with APBI. Low maximum skin dose and the small high-dose volumes are crucial in maintaining good cosmetic outcomes [2, 26–30]. In HDR interstitial brachytherapy volumes receiving 150 and 200 % of prescription dose have been shown to correlate with toxicity [31]. Because of the link between toxicity and dosimetric parameters, the NSABP B39/RTOG 04-13 requires V_150_ to be ≤ 70 cm^3^ and V_200_ to be less than 20 cm^3^ in multi-catheter treatment and V_150_ to be ≤ 50 cm^3^ and V_200_ to be less than 10 cm^3^ in MammoSite balloon treatment.

It has been established that multi-lumen applicators allow for better optimization of dose distribution in the treatment area, minimizing the risk to nontarget areas [32, 31, 33]. However, this was not always the case in our data set as multi-lumen balloon applicators showed no statistically significant advantage to single-lumen balloon applicators when comparing D_max_Skin or D_max_Rib. However, multi-lumen balloon applicators were able to achieve smaller high-dose volumes when compared to single-lumen balloon applicators.

Strut-based intracavitary devices showed a clear advantage in D_90_, D_max_Skin, and D_max_Rib, when compared to balloon-based applicators. Higher maximum doses in balloon applicators could be from confluent "hot spots" seen at the balloon surface [3]. Instead of a balloon, SAVI devices have a central catheter that is surrounded by multiple struts containing multiple dwell positions for the radioactive source. This design allows preferential treatment to the side of the cavity closest to the surgical margin and eliminates balloon surface "hot spots" [22]. The unique and flexible design could also account for the better D_90_ and D_95_. Additionally, in balloon-based applicators, seroma formation has been shown to be a function of radiation hot spots [34, 23].

Strut-based devices were significantly better at keeping the D_max_Skin and D_max_Rib, under the 125 and 100 % thresholds. This may result in less skin and rib toxicity. Higher doses to the ribs are associated with fractures and limiting this value can decrease its incidence [14]. Vargo et. al recently showed telangiectasia development to be a function of skin dose, where a skin dose >100 % was the strongest predictor for telangiectasia development [23]. Cuttino et al. also showed that the maximal dose delivered to the skin was significantly associated with the incidence of telangiectasia and moderate to severe fibrosis, especially when doses were >120 % of the prescription dose [3]. The higher D_max_Skin in single-lumen devices was expected as dose constraints are sometimes not feasible as single-channel balloon catheters may thin out the anterior tissue plane [35].

Our data was similar to Patel et. al, who showed strut-based intracavitary implants are associated with a significantly greater V_200_ and smaller V_150_ when compared to balloon implants [36]. A suboptimal cosmetic outcome and skin toxicity has been shown to be significantly associated with V_150_ and V_200_, and inversely related to the dose homogeneity index which is defined as (1 – V_150_/V_100_). The development of fat necrosis has also been shown to be associated with V_150_ and V_200_, while late subcutaneous toxicity has only shown associations with dose homogeneity index [31]. Even though clinical significance has not yet been established, the dose homogeneity index with SAVI is typically lower than those seen in interstitial or balloon brachytherapy [22]. V_150_ and V_200_ could also have to reach a specific threshold to result in toxicity. Cuttino et. al saw no association between outcomes and V_150_ or V_200_, but accounted for this because only 4 % of their patient population exceeded a V_200_ of 20 cm^3^ [3]. Our results were similar, as only one patient (0.1 %) exceeded a V_200_ of 20 cm^3^.

Because strut-based devices were used on smaller PTVs we wanted to see how the PTV affected dosimetric parameters. The only strong correlation in all devices was seen between PTV and V_150_ showing that with greater treatment volumes higher dose volumes should be expected. PTV and V200 also showed a strong correlation in SAVI-mini devices. PTV was weakly negatively correlated with max skin dose and max rib dose and weakly positively correlated with D_95_ across the strut based and multi-channel balloon applicators. Because of this weak correlation it is hard to anticipate maximum doses to the skin and ribs by PTV alone. While our study had a minimum skin bridge of 5 mm for all patients, based on the correlation between smaller PTV and maximum skin dose the SAVI-mini may prove to be appropriate for those with less than 5 mm of skin distance. The variable of skin bridge was eliminated because we had a cutoff of 5 mm. No patients experienced a fracture within the follow-up period within the contoured rib structures.

This study has several limitations. Because this study was a retrospective analysis there was a lack of standardization among patients. The group of patients receiving strut-based devices was also much larger than the patients receiving single-lumen or multi-lumen balloon applicators.

## Conclusion

The data from this study shows PTV volume is strongly correlated with V_150_ in all devices and V_200_ in strut based devices. Larger PTV volumes result in greater V_150_ and V_200_, which could help predict potential risks for hotspots and resulting toxicities in these devices. PTV volume is also weakly negatively correlated with max skin dose and max rib dose, meaning that as the PTV volumes increase one can expect slightly smaller max skin and rib doses. Strut based applicators are significantly more effective in keeping skin and rib dose constraints under 125 and 100 % when compared to any balloon based applicator and also achieve a significantly better D_90_.
